# Heat shock protein 70/peptide complexes: potent mediators for the generation of antiviral T cells particularly with regard to low precursor frequencies

**DOI:** 10.1186/1479-5876-9-175

**Published:** 2011-10-12

**Authors:** Sabine Tischer, Megan Basila, Britta Maecker-Kolhoff, Stephan Immenschuh, Mathias Oelke, Rainer Blasczyk, Britta Eiz-Vesper

**Affiliations:** 1 Institute for Transfusion Medicine, Hannover Medical School, Hannover, Germany; 2 Integrated Research and Treatment Center (IFB-Tx), Hannover Medical School, Hannover Germany; 3 Department of Pediatric Hematology/Oncology, Hannover Medical School, Hannover, Germany; 4 Department of Pathology and Medicine, Johns Hopkins School of Medicine, Baltimore, Maryland, USA

## Abstract

**Background:**

Heat shock protein 70 (HSP70) has gained major attention as an adjuvant capable of inducing antigen-specific CD8^+ ^and CD4^+ ^T-cell responses. The ability of HSP70/peptide complexes to elicit cytotoxic T-cell (CTL) responses by cross-presentation of exogenous antigens via HLA class I molecules is of central interest in immunotherapy. We examined the role of HSP70/CMVpp65_495-503_-peptide complex (HSP70/CMV-PC) in HLA class I-restricted cross-presentation for *ex vivo *expansion of CMV-specific CTLs.

**Methods:**

CMV-specific T cells generated from PBMCs of HLA-A*02:01/CMV-seropositive donors were stimulated for 21 days with HSP70/CMV-PC and analyzed in functional assays. As a control PBMCs were cultured in the presence of CMVpp65_495-503 _peptide or HSP70. Increase of CMV-specific CTLs was visualized by pentameric HLA-A*02:01/CMVpp65_495-503 _complex.

**Results:**

About 90% of HSP70/CMV-PC generated T cells were CMV-specific and exhibited significantly higher IFN-γ secretion, cytotoxic activity, and an increased heme oxygenase 1 (HO-1) gene expression as compared to about 69% of those stimulated with CMVpp65_495-503 _peptide. We decided to classify the HLA-A*02:01/CMV-seropositive donors as weak, medium, and strong responder according to the frequency of generated A2/CMV-pentamer-positive CD8^+ ^T cells. HSP70/CMV-PC significantly induces strong antiviral T-cell responses especially in those donors with low memory precursor frequencies. Blockage of CD91 with α2-macroglobulin markedly reduced proliferation of antiviral T cells suggesting a major role of this receptor in the uptake of HSP70/CMV-PC.

**Conclusion:**

This study clearly demonstrates that HSP70/CMV-PC is a potent mediator to induce stronger T-cell responses compared to antiviral peptides. This simple and efficient technique may help to generate significant quantities of antiviral CTLs by cross-presentation. Thus, we propose HSP70 for chaperoning peptides to reach an efficient level of cross-presentation. HSP70/peptide complexes may be particularly useful to generate stronger T-cell responses in cases of low precursor frequencies and may help to improve the efficiency of antigen-specific T-cell therapy for minor antigens.

## Background

Heat shock proteins (HSPs) are highly conserved proteins that function primarily as intracellular molecular chaperones. Because of their ability to interact with proteins and peptides, they play an important role in cell and organ survival [1]. HSPs play a key role in protein degradation, intracellular transport processes, protein folding, and antigen processing. In apoptotic pathways, they act at multiple points to prevent cells from inappropriate cell death triggered by stress-induced damage [2]. The observation that tumor-derived preparations of HSPs, such as glucose-regulated protein 96 (gp96), HSP70, and HSP90 can elicit specific anti-tumor T-cell immune responses, suggests that heat shock proteins might have immunotherapeutic potential [3,4]. This possibility is currently under investigation in clinical trials [5-7].

The immunogenicity of HSP preparations is caused by the binding of antigenic peptides to HSPs and from the transfer of these peptides to professional antigen-presenting cells (APCs), such as dendritic cells (DCs). Peptides chaperoned by HSPs are taken up in a receptor-dependent manner and channeled into the major histocompatibility complex (MHC) class I processing pathway for loading onto MHC class I molecules and subsequent presentation to CD8^+ ^cytotoxic T lymphocytes (CTLs) [8,9]. Additionally, heat shock proteins and HSP/peptide complexes (HSP/PCs) can stimulate the maturation of APCs [10-12] by efficiently interacting with receptors [13] such as CD91 [14,15], toll-like receptor (TLR) 2, TLR4 [16,17], Lox-1 [18], or CD40 [19].

Various groups have reported that *in vitro *generated tumor-derived HSP/PCs are potent adjuvants to facilitate the presentation of tumor antigens and the induction of anti-tumor immunity [3,4]. However, it is widely unknown whether viral peptides chaperoned by human HSPs are sufficiently capable of cross-priming CD8^+ ^antiviral T cells [20]. Viral infections resulting from reactivation of latent viruses such as cytomegalovirus (CMV), human adenovirus (ADV), and Epstein-Barr virus (EBV) are associated with high morbidity and mortality after hematopoietic stem cell transplantation (HSCT) [21-24] and solid organ transplantation [25-27]. Antiviral agents such as ganciclovir can reduce the incidence of early viral diseases, but are associated with substantial toxicity and may result in delayed immune reconstitution [28]. Previous studies have shown that adoptive immunotherapy with donor-derived virus-specific CTLs generated *in vitro *can safely and efficiently prevent the clinical manifestation of these viral diseases in patients following transplantation with no acute toxicities or increased risk of graft-versus-host disease (GvHD) [21,23,24,28].

In this study, we investigated whether an HSP/PC consisting of HSP70 plus the immunodominant HLA-A*02:01-restricted CMV peptide (CMVpp65_495-503_) [29] can enhance cross-presentation of MHC class I molecules and may therefore result in a higher specific antiviral T-cell response compared to the stimulation with the peptide alone. Most protocols for *ex vivo *activation and expansion of antiviral T cells for adoptive immunotherapy use either peptide-loaded DCs, artificial APCs (aAPCs), or CMV-infected immature DCs as stimulator cells [30-33]. Additionally, researchers have focused on the whole CMVpp65 protein, whole viral lysates, virally infected cells, and various HLA-restricted viral peptides as a source of immunodominant antigens stimulating both CTLs and T helper (Th) cells [21,29,30,34]. The present study demonstrates that cross-presentation of viral antigens by lipopolysaccharide-(LPS) free HSP70 [35] significantly increases the efficiency of the antiviral T-cell response. Our findings highlight the role of extracellular HSP70 in the activation of the adaptive immune response. The described method for *in vitro *preparation of the HSP70/CMVpp65_495-503_-peptide complex (HSP70/CMV-PC) and the generation of CMV-specific CTLs can be adapted to GMP conditions and used for therapeutic applications.

## Methods

### Preparation of HSP70/CMVpp65_495-503_-peptide complex (HSP70/CMV-PC)

To facilitate eukaryotic expression and isolation, we developed an expression strategy for soluble human HSP70 secreted into the cell culture supernatant of mammalian cells [35]. Purified endotoxin-free HSP70 was used to prepare HSP70/CMV-PC under conditions similar to those described elsewhere [36,37]. Briefly, 10 μg of HSP70 was incubated at 37°C for 2 h with and without a 150-fold molar excess of CMVpp65_495-503 _peptide (NLVPMVATV, purity > 95%, Eurogentec, Seraing, Belgium) plus HSP70-peptide-binding buffer (PBS with 1 mM ADP, 1 mM MgCl_2_, pH 7.4) to yield a total volume of 100 μl. After adding 2 ml PBS to the complex solution non-conjugated peptide was removed completely by filtration through a 30 kDa molecular weight cutoff filter unit (Millipore, Schwalbach, Germany). The final concentration of HSP70/CMV-PC was determined by Bradford protein analysis. To verify the concentration of free, uncomplexed peptide remained in the spin column flow, additional independent experiments (n = 5) were performed using fluorescein isothiocyanate (FITC)-labeled CMVpp65_495-503 _peptide ((NLVPMK[FITC]VATV; CMV[FITC], purity > 95%, GL Biochem, Shanghai, China) for preparation. After washing the recovery of HSP70/CMV[FITC]-PC was determined on a Synergy 2 Microplate Reader (Bio Tek Instruments, Winooski, USA). The effective concentration of peptide bound to HSP70 was calculated as the starting amount (20 μg) minus the amount in the flow through. This count corresponds to a loading efficiency of ~48% for the (FITC)-labeled peptide on HSP70, which correspond to the generation of about 10 μg/ml HSP70/CMV-PC.

### Stimulation of antiviral T cells with HSP70, CMVpp65_495-503 _peptide, and HSP70/CMV-PC

Baseline frequencies of CMV-specific T cells were determined prior to stimulation. Briefly, blood from 50 healthy HLA-A*02:01/CMV-seropositive platelet donors with no prior history of blood transfusion was stained with the R-phycoerythrin (R-PE)-conjugated Pro5 pentamer HLA-A*02:01/CMVpp65_495-503 _(A2/CMV-pentamer, Proimmune, Oxford, UK) and fluorescein isothiocyanate (FITC)-conjugated anti-CD8 monoclonal antibody, allophycocyanin (APC)-conjugated monoclonal antibody, and peridinin chlorophyll protein (PerCP)-conjugated monoclonal antibody (mAB, BD Biosciences, Heidelberg, Germany), respectively. 100 μl whole blood samples were then washed with 2 ml PBS and stained with A2/CMV-pentamer and anti-CD8 antibody. After adding 2 ml ammonium chloride lysis solution, the cells were incubated for 10 min, washed twice with PBS and analyzed on a flow cytometer (FACSCanto, BD Biosciences, Heidelberg, Germany).

T-cell stimulation was performed using samples from 16 A2/CMV-pentamer-positive donors. Peripheral blood mononuclear cells (PBMCs) were isolated by discontinuous gradient centrifugation, washed twice in sterile PBS, resuspended at a concentration of 1 × 10^7 ^cells/ml in RPMI1640 culture medium (Lonza, Vervies, Belgium), and then supplemented with 10% heat-inactivated human AB serum (C.C.pro, Neustadt, Germany) and 100 U/ml IL-2 (PeproTech, Hamburg, Germany). Cells were stimulated in a 24-well plate with either 10 μg/ml HSP70, 10 μg/ml CMVpp65_495-503 _peptide, or 10 μg/ml HSP70/CMV-PC. HSP70-peptide-binding buffer was used as negative control.

On days 7, 14, and 21, supernatants were harvested for granzyme B and IFN-γ secretion analyses by ELISA, and the T cells were restimulated with autologous PBMCs pulsed with the peptide or the complex. Briefly, 1 × 10^7 ^autologous PBMCs/ml were pulsed with HSP70, CMVpp65_495-503 _peptide, or HSP70/CMV-PC overnight in serum-free RPMI1640 medium. 2.5 × 10^5 ^T cells were restimulated with irradiated (30 Gy) PBMCs pulsed with the peptide or the complex at a responder-to-stimulator ratio of 10:1 in culture media containing 10% AB serum and 100 U/ml IL-2 (96-well round-bottom plate, 200 μl) for a total of two restimulation cycles.

In order to determine the frequency of induced antiviral T cells, the cells were stained weekly with the A2/CMV-pentamer and the following mAbs: PerCP-conjugated anti-CD8, FITC-conjugated anti-CD25, FITC-conjugated anti-CD69, and APC-conjugated anti-CD137 (all from BD Biosciences). All samples were analyzed on a flow cytometer with live gating on lymphocytes during acquisition. For analysis, cells were gated on either CD8^+ ^T cells or A2/CMV-pentamer-positive CD8^+ ^T cells. mRNA levels of heme oxygenase 1 (HO-1, also referred to as HSP32) and HSP70 were assessed every second day after stimulation/restimulation and, finally, on day 21.

### Determination of cytolytic activity

Cytolytic activity of induced antiviral T cells was determined once weekly after the first (day 14) and second restimulation cycle (day 21) in a non-radioactive flow cytometric assay using autologous CFSE [5- or 6-(N-succinimidyloxicarbonyl)-3',6'-O,O'-diacetylfluorescein)]-labeled CMVpp65_495-503 _peptide-loaded autologous PBMCs [38] as target cells. In order to exclude alloreactivity the generated T cells were also tested against unloaded CFSE-labeled PBMCs. Briefly, T cells were incubated with target cells in 96-well round-bottom plates at an effector to target cell ratio (E:T) of 10:1 and 1:1 in the presence of 20 U/ml IL-2 (PeproTech). Target cell lysis was assessed by 7-aminoactinomycin D (7-AAD, BD Biosciences) staining after 5 h.

### T-cell proliferation assay and blocking of HSP70/CMV-PC uptake by addition of a2-macroglobulin

To observe the expression levels of CD91 and CD40 we stained freshly isolated PBMCs with PE-conjugated anti-CD91 and PE-conjugated anti-CD40 (AbD Serotec, NC, USA) and analyzed them by flow cytometry.

PBMCs from 5 A2/CMV-pentamer-positive donors were labeled with CFSE (Invitrogen, Karlsruhe, Germany) at a final concentration of 4 μM and plated at 5 × 10^5 ^cells per well (96-well round-bottom plate) in 200 μl RPMI1640/10% AB serum containing 100 U/ml IL-2. Cells were stimulated for 4 days with either 10 μg/ml HSP70, 10 μg/ml CMVpp65_495-503 _peptide, or 10 μg/ml HSP70/CMV-PC. Unstimulated T cells were used as negative control.

To determine whether antiviral T-cell induction occurred due to cross-presentation of peptides by HSP70, the uptake of HSP70/CMV-PC was blocked by adding 500 μg/ml α2-macroglobulin (A2M, BioMac, Leipzig, Germany), the natural ligand of CD91. Therefore, 5 × 10^5 ^CFSE-labeled PBMCs were pulsed prior to stimulation with 500 μg/ml A2M for 1 hour and then stimulated for 4 days with 10 μg/ml HSP70, CMVpp65_495-503 _peptide, or HSP70/CMV-PC. As negative control unstimulated A2M-pulsed unstimulated T cells were used. T cells were stained with APC-conjugated anti-CD8 mAb and R-PE-conjugated A2/CMV-pentamer and the effect of stimulation with HSP70, CMVpp65_495-503 _peptide, and HSP70/CMV-PC with or without A2M on CD8^+ ^and A2/CMV-pentamer-positive CD8^+ ^T-cell proliferation was analyzed by flow cytometry.

### Determination of HSP70 and HO-1 mRNA levels by quantitative RT-PCR

HSP70 and HO-1 mRNA levels were determined every second day after stimulation/restimulation and, finally, on day 21. Total cellular RNA was isolated (RNeasy Mini Kit, Qiagen) and cDNA amplified using the High Capacity cDNA Reverse Transcription Kit (Applied Biosystems, Darmstadt, Germany) for this purpose. Inventoried mixes (Applied Biosystems) were used for quantification of HSP70 and HO-1 mRNA levels. Amplification was performed using TaqMan Gene Expression Master Mix (Applied Biosystems). Thermal cycling was performed on a StepOnePlus real-time PCR system (Applied Biosystems) at 50°C for 15 min and 95°C for 10 min followed by 40 cycles at 95°C for 15 sec and 60°C for 1 min. The constitutively expressed GAPDH gene was used as the reference standard for normalization of mRNA levels.

### Determination of granzyme B and IFN-γ secretion by ELISA

Granzyme B and IFN-γ secretion in the supernatant of cells cultured in the presence of HSP70-peptide-binding buffer (negative control), HSP70, CMVpp65_495-503 _peptide, or HSP70/CMV-PC was measured on days 7, 14, and 21. Granzyme B (Bender MedSystems, Vienna, Austria) and IFN-γ (eBioscience, San Diego, USA) ELISAs were performed according to the manufacturer's instructions.

### Statistics

Statistical analyses were performed using t-test run on GRAPHPAD PRISM V5.02 software (GraphPad Software, San Diego, California, USA). Levels of significance are expressed as p-values (*p < 0.05, **p < 0.01, ***p < 0.001).

## Results

### Induction and expansion of antiviral T-cell populations in response to HSP70, CMVpp65_495-503 _peptide, and HSP70/CMV-PC

We have previously developed a method for the expression of endotoxin-free recombinant human HSP70 [35], which was used in this study to chaperone viral peptides into the MHC class I cross-presentation pathway to increase the efficiency of the antiviral T-cell response. Human HSP70 preparations containing less than 1 EU/ml endotoxin were defined as endotoxin-free.

To directly visualize CMV-specific T cells in the blood of healthy donors before stimulation, we stained whole blood from 50 HLA-A*02:01/CMV-seropositive donors with the A2/CMV-pentamer and anti-CD8 antibody. The pentamer bound 0.30% to 6.70% (mean 1.91%) of CD8^+ ^T cells in 26/50 (52.00%) HLA-A*02:01-positive donors. No A2/CMV-pentamer-positive T cells (< 0.30% A2/CMV-pentamer^+^CD8^+ ^T cells) were detected in 24 donors (48.00%). PBMCs from sixteen A2/CMV-pentamer-positive donors (mean 1.62%, range 0.30%-5.64%) were selected for further analysis (Table 1). The frequency and activation status of antiviral T cells stimulated with human HSP70, CMVpp65_495-503 _peptide, or HSP70/CMV-PC, respectively, were assayed weekly by determining the percentage of T cells stained positive for A2/CMV-pentamer, CD8, CD4, CD25, CD69, and CD137, respectively. Table 2 shows the frequencies of CD8^+ ^T cells on day 0 and after 7, 14, and 21 days of stimulation under the described conditions.

**Table 1:** Classification of the three responder groups (weak, medium, and strong) after stimulation with CMVpp65_495-503 _peptide.

	day 0		day 7		
	
donor	percentage of pentamer^+^CD8^+ ^T cells	absolute number of pentamer^+^CD8^+ ^T cells [x10^6^]	percentage of pentamer^+^CD8^+ ^T cells	absolute number of pentamer^+^CD8^+ ^T cells [x10^6^]	responder classification
1	0.40	0.04	0.30	0.02	weak
2	0.39	0.04	0.19	0.02	weak
3	0.39	0.04	0.34	0.06	weak
4	0.37	0.04	0.50	0.07	weak
5	0.40	0.04	0.14	0.02	weak
6	0.30	0.03	4.50	0.56	medium
7	0.53	0.05	5.19	0.36	medium
8	2.17	0.22	2.75	0.17	medium
9	0.79	0.08	2.89	0.38	medium
10	0.30	0.03	1.11	0.19	medium
11	0.65	0.07	37.76	4.34	strong
12	5.64	0.56	13.54	1.15	strong
13	0.56	0.06	21.00	1.26	strong
14	1.40	0.14	16.67	2.17	strong
15	0.70	0.07	16.80	1.60	strong
16	1.70	0.17	19.50	1.37	strong

The data sets from 16 healthy HLA-A*02:01/CMV-seropositive donors were divided into 3 responder groups: weak (n = 5), medium (n = 5), and strong (n = 6), respectively, according to the obtained frequency of generated A2/CMV-pentamer-positive CD8^+ ^T cells after 7 days of stimulation with the CMVpp65_495-503 _peptide (weak responders < 1% A2/CMV-pentamer-positive CD8^+ ^T cells, medium responders 1-10% A2/CMV-pentamer-positive CD8^+ ^T cells, strong responders > 10% A2/CMV-pentamer-positive CD8^+ ^T cells).

**Table 2:** Antiviral T-cell frequencies generated by the different stimuli.

**A**. **Percentage of CD8^+ ^T cells^# ^**			
**CD8^+ ^T cells **(day 0: 20.22 ± 1.89)			
	**day 7**	**day 14**	**day 21**

NC	25.48 ± 1.93	33.80 ± 20.15	41.92 ± 23.91
HSP70	25.20 ± 2.50	36.52 ± 12.65	47.70 ± 28.96
CMVpp65_495-503 _peptide	27.07 ± 1.22	61.43 ± 11.61	63.06 ± 25.79
HSP70/CMV-PC	27.27 ± 0.95	79.83 ± 11.36**	83.17 ± 5.30*

^#^cells were gated on viable lymphocytes			
**B. Percentage of CD25^+^, CD69^+^, and CD137^+ ^T cells^□^**			

**CD25^+ ^T cells **(day 0: 1.29 ± 0.21)			
	**day 7**	**day 14**	**day 21**

NC	0.17 ± 0.29	0.23 ± 0.10	0.08 ± 0.04
HSP70	0.13 ± 0.23	0.28 ± 0.15	0.18 ± 0.08
CMVpp65_495-503 _peptide	0.77 ± 0.29	36.18 ± 13.12	5.40 ± 5.29
HSP70/CMV-PC	1.74 ± 0.81	38.25 ± 8.16	18.10 ± 0.57*

**CD69^+ ^T cells **(day 0: 13.62 ± 4.70)			

NC	0.10 ± 0.10	0.23 ± 0.10	0.18 ± 0.18
HSP70	0.10 ± 0.10	0.30 ± 0.14	0.24 ± 0.27
CMVpp65_495-503 _peptide	0.73 ± 0.22	2.68 ± 1.18	3.68 ± 4.18
HSP70/CMV-PC	1.33 ± 0.59	2.18 ± 0.35	5.82 ± 7.71

**CD137^+ ^T cells **(day 0: 2.39 ± 0.01)			

NC	0.10 ± 0.10	0.10 ± 0.08	0.04 ± 0.05
HSP70	0.07 ± 0.06	0.15 ± 0.06	0.14 ± 0.09
CMVpp65_495-503 _peptide	0.70 ± 0.17	12.77 ± 4.24	6.60 ± 1.39
HSP70/CMV-PC	1.50 ± 0.52*	25.10 ± 4.67*	14.15 ± 5.87*

^□^cells were gated on CD8^+ ^T cells			
**C. Percentage of CD25^+^, CD69^+^, and CD137^+ ^T cells^○^**			

**CD25^+ ^T cells **(day 0: 5.98 ± 0.33)			
	**day 7**	**day 14**	**day 21**

NC	51.03 ± 44.41	49.46 ± 43.74	51.03 ± 56.44
HSP70	49.57 ± 43.27	48.62 ± 45.50	50.50 ± 55.42
CMVpp65_495-503 _peptide	87.37 ± 2.90	72.90 ± 25.77	73.03 ± 45.76
HSP70/CMV-PC	89.50 ± 5.40	78.55 ± 24.43	72.40 ± 47.37

**CD69^+ ^T cells **(day 0: 16.49 ± 10.89)			

NC	5.45 ± 4.74	10.73 ± 4.10	2.63 ± 4.56
HSP70	5.85 ± 3.89	12.08 ± 6.67	6.17 ± 8.45
CMVpp65_495-503 _peptide	84.30 ± 0.52	2.93 ± 1.31	0.50 ± 0.44
HSP70/CMV-PC	84.93 ± 12.00	3.07 ± 1.24	0.63 ± 0.58

**CD137^+ ^T cells **(day 0: 11.86 ± 1.01)			

NC	2.60 ± 2.40	4.23 ± 3.10	3.30 ± 1.87
HSP70	4.00 ± 3.82	8.45 ± 4.63	4.83 ± 3.61
CMVpp65_495-503 _peptide	88.05 ± 3.04	15.45 ± 7.19	10.73 ± 8.39
HSP70/CMV-PC	92.45 ± 5.87	17.05 ± 14.79	11.45 ± 9.73

**^○^**cells were gated on A2/CMV-pentamer-positive CD8^+ ^T cells

We assessed the capacity of T-cell subpopulations from five A2/CMV-pentamer-positive healthy donors to generate antiviral T cells in the presence of HSP70, CMVpp65_495-503 _peptide, or HSP70/CMV-PC by enumerating the following specific T cells by flow cytometry. (A) CD8^+ ^T cells gated on viable lymphocytes, (B) CD25^+^, CD69^+^, and CD137^+ ^cells gated on CD8^+ ^T cells and (C) CD25^+^, CD69^+^, and CD137^+ ^cells gated on A2/CMV-pentamer-positive CD8^+ ^T cells. PBMCs cultured in the presence of HSP70-peptide-binding buffer were used as negative controls (NC). The results of 5 independent experiments using cells from medium responder are expressed as mean ± SD. Asterisks shown indicate only statistically significant differences between levels in CMVpp65_495-503 _peptide- and HSP70/CMV-PC-stimulated cells (* p < 0.05, ** p < 0.01 or *** p < 0.001).

**Figure 1:**
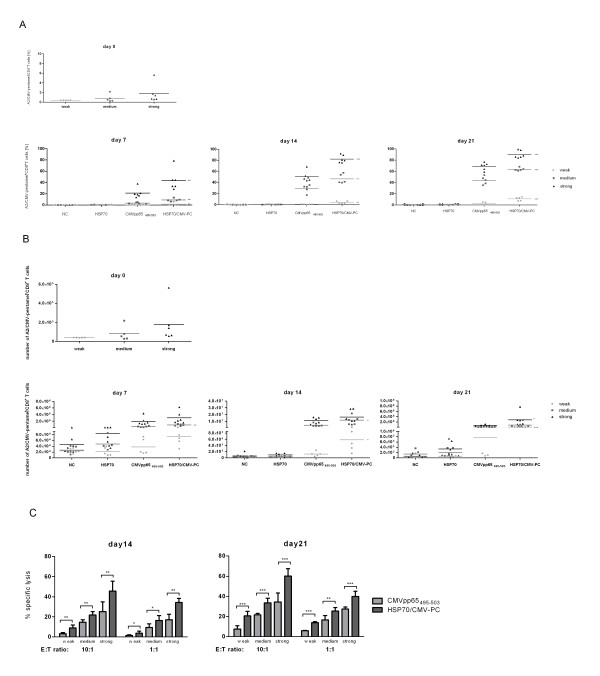
HLA-A*02:01/CMV-pentamer staining, number of A2/CMV-pentamer-positive CD8^+ ^T cells, and cytolytic function of antigen-specific CTLs stimulated with HSP70/CMV-PC and CMVpp65_495-503 _peptide. Frequency (A) and number (B) of HLA-A*02:01/CMV-pentamer-positive CD8^+ ^CTLs from 16 healthy HLA-A*02:01/CMV-seropositive platelet donors on day 0 and 7, 14, and 21 days after stimulation with recombinant HSP70, CMVpp65_495-503 _peptide, or HSP70/CMV-PC. Cells cultured in the presence of the HSP70-peptide-binding buffer served as negative controls (NC). The 16 donors were divided into three groups (weak: n = 5, medium: n = 5, strong: n = 6) according to the frequency of generated A2/CMV-pentamer-positive CD8^+ ^T cells on day 7. Cytolytic activity (C) of expanded T cells against antigen-loaded PBMCs after one (day 14) and two (day 21) restimulation cycles in cells from 16 healthy HLA-A*02:01/CMV-seropositive donors. CFSE-labeled PBMCs unloaded or loaded with CMVpp65_495-503 _peptide were used as target cells. T cells generated for 14 or 21 days in the presence of the CMVpp65_495-503 _peptide or HSP70/CMV-PC (effector cells) were co-cultured with target cells for 5 h at a cell ratio of 10:1 or 1:1, respectively. The basal cytotoxic activity of effector T cells induced by CMVpp65_495-503 _peptide or HSP70/CMV-PC against the unloaded target cells was subtracted from the cytotoxic values measured after incubation of effector T cells against CMVpp65_495-503 _peptide-loaded PBMCs. The results of independent experiments are expressed as mean ± SD. Asterisks shown in the Figure indicate only statistically significant differences between levels in CMVpp65_495-503 _peptide- and HSP70/CMV-PC-stimulated cells (* p < 0.05, ** p < 0.01, *** p < 0.001).

The highest frequencies of CD8^+ ^T cells were observed on days 7 to 21 in cells stimulated with the complex with means ranging from 27.27% ± 0.95 to 83.17% ± 5.30 (Table 2). Data sets from 16 healthy HLA-A*02:01/CMV-seropositive donors were divided into 3 responder groups: weak, medium, and strong, respectively (Figure 1A and 1B, Table 1), according to the obtained frequency of generated A2/CMV-pentamer-positive CD8^+ ^T cells after 7 days of stimulation with the CMVpp65_495-503 _peptide. Classification of groups was performed as followed: the group of weak responders included 5 donors with < 1% A2/CMV-pentamer-positive CD8^+ ^T cells (mean 0.29%, range 0.14%-0.50%) and the group of medium responders contained 5 donors with 1-10% A2/CMV-pentamer-positive CD8^+ ^T cells (mean 3.29%, range 1.11%-5.19%). The group of strong responders consisted of 6 donors with > 10% A2/CMV-pentamer-positive CD8^+ ^T cells (mean 20.88%, range 13.54%-37.76%) after one week of stimulation.

Flow cytometric results of the stimulation experiments for one representative donor from each group are shown (Additional file 1). Both the peptide and the complex significantly increased populations of A2/CMV-pentamer-positive CD8^+ ^T cells in all donors tested (Figure 1A and 1B), whereas HSP70 or HSP70-peptide-binding buffer alone (negative controls) have no effect on T-cell induction. As shown in Figure 1A, on day 7 the number of A2/CMV-pentamer-positive CD8^+ ^T cells induced by the complex (weak: mean 0.56%, medium: mean 9.22%, strong: mean 43.78%) was higher than those induced by the peptide alone (weak: mean 0.29%, medium: mean 3.29%, strong: mean 20.88%). On day 14 the A2/CMV-pentamer-positive CD8^+ ^T-cell frequencies induced by the complex (weak: mean 4.09%, medium: mean 46.68%, strong: mean 82.35%) were significantly higher than those achieved by the peptide alone (weak: mean 0.69%, medium: mean 29.20%, strong: mean 50.80%). The highest frequencies of A2/CMV-pentamer-positive CD8^+ ^T cells (weak: mean 11.03%, medium: mean 63.57%, strong: mean 90.08%) were observed on day 21 in cells treated with HSP70/CMV-PC, which were significantly higher than those induced by the peptide alone (weak: mean 2.32%, medium: mean 44.02%, strong: mean 69.12%).

The frequencies of A2/CMV-pentamer-positive CD8^+ ^T cells correlate with the obtained cell numbers of A2/CMV-pentamer-positive CD8^+ ^T cells (Figure 1B). From day 0 to day 21 the increase in A2/CMV-pentamer-positive CD8^+ ^T cells induced by the complex (weak: mean 3.90 × 10^4 ^to 3.82 × 10^6 ^/97.95-fold, medium: mean 8.12 × 10^4 ^to 8.12 × 10^7 ^/1000-fold, strong: mean 1.78 × 10^5 ^to 2.78 × 10^8 ^/1564.3-fold) was significantly higher than those achieved by the peptide alone (weak: mean 3.90 × 10^4 ^to 7.60 × 10^5 ^/19.5-fold, medium: mean 8.12 × 10^4 ^to 1.10 × 10^7 ^/135.4-fold, strong: mean 1.78 × 10^5 ^to 6.81 × 10^7 ^/383.5-fold).

To sum up, from day 7 forth the highest frequencies and cell numbers of A2/CMV-pentamer-positive CD8^+ ^T cells were induced by HSP70/CMV-PC. Especially in donors with low memory precursor frequencies, the stimulation with the complex resulted in a significantly higher level of antigen-specific CD8^+ ^T cells compared to the stimulation with the viral peptide alone.

Because CD25, CD69, and CD137 are suitable surface markers to differentiate antigen-specific T cells [39], they were used to further characterize CD8^+ ^(Table 2) and A2/CMV-pentamer-positive CD8^+ ^T cells (Table 2). Expression of all three markers increased after 14 days in CD8^+ ^T cells. As expected, expression levels of all markers in A2/CMV-pentamer-positive CD8^+ ^T cells were higher than those in CD8^+ ^T cells. Furthermore, the highest expression levels were observed in all A2/CMV-pentamer-positive CD8^+ ^populations on day 7 which significantly decreases up to day 21. Expression levels were slightly higher in CD8^+ ^T cells and A2/CMV-pentamer-positive CD8^+ ^T cells, respectively stimulated with HSP70/CMV-PC as compared to those stimulated with CMVpp65_495-503 _peptide alone.

### Cytolytic activity of induced antiviral T cells

To examine whether induced antigen-specific T cells were functional, a non-radioactive cytotoxicity assay was performed. Cytolytic activity of CMVpp65_495-503 _peptide- and HSP70/CMV-PC-induced CTLs from 16 healthy HLA-A*02:01/CMV-seropositive donors were analyzed after one and two restimulation cycles (Figure 1C). As a control T cells were cultured in the presence of HSP70 or HSP70-peptide-binding buffer. The lytic function of the CTLs was assayed by E:T cell ratios of 10:1 and 1:1. CFSE-labeled PBMCs pulsed with CMVpp65_495-503 _peptide were used as target cells. Additionally to exclude unspecific cytolytic function of the effector cells, non-pulsed PBMCs were used as target cells as well. The basal cytolytic activity of effector T cells against the non-pulsed target cells was subtracted from the specific cytolytic values. The results are expressed as the mean percentage of target cell lysis ± standard deviation. In all three responder groups, unstimulated T cells and HSP70-induced T cells exhibited the lowest levels of cytolytic activity (data not shown). The specific lysis of CMVpp65_495-503 _peptide-pulsed target cells by induced antiviral CTLs increased from the first to the second restimulation cycle at both E:T ratios.

The highest cytolytic activity was observed for HSP70/CMV-PC-induced CTLs at an E:T ratio of 10:1 on day 14 (weak: 9.05%, medium: 21.71%, strong: 45.52%) and on day 21 (weak: 20.62%, medium: 33.41%, strong: 60.12%). The killing was significant higher compared CMVpp65_495-503 _peptide-induced CTLs (weak: 3.25% and 7.55%, medium: 14.65% and 21.64%, strong: 25.14 and 34.37%). Co-culturing at an E:T ratio of 1:1 showed similar results. In summary, in all three groups the cytolytic activity of T cells stimulated with HSP70/CMV-PC was significantly higher than that of CTLs stimulated with the CMVpp65_495-503 _peptide alone. These findings indicate that T cells stimulated with HSP70/CMV-PC are cytolytically active and recognize endogenously processed CMVpp65 antigen-HLA complexes more effectively than T cells stimulated with peptide alone.

### Inhibition of T-cell proliferation and blockage of HSP70/CMV-PC uptake by A2M

**Figure 2:**
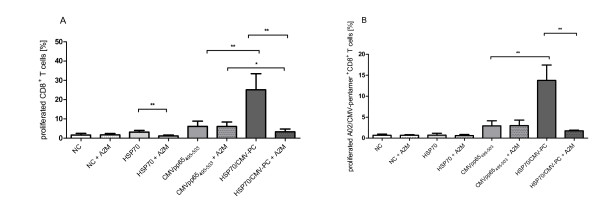
**Effects of HSP70, CMVpp65_495-503 _peptide, and HSP70/CMV-PC in the presence/absence of α2-macroglobulin on T-cell proliferation**. PBMCs were labeled with CSFE and cultured with HSP70-peptide-binding buffer (NC), HSP70, CMVpp65_495-503 _peptide, or HSP70/CMV-PC for 4 days. Cell proliferation within the CD8^+ ^(A) and the A2/CMV-pentamer-positive CD8^+ ^(B) T-cell populations was evaluated by CFSE dilution. In order to determine whether antiviral T-cell induction occurred due to cross-presentation of peptides by HSP70, the uptake of HSP70/CMV-PC was blocked by adding α2-macroglobulin (A2M). The results of 5 independent experiments using cells from medium responder are expressed as mean ± SD. Asterisks shown in the Figure indicate only statistically significant differences between levels in CMVpp65_495-503 _peptide- and HSP70/CMV-PC-stimulated cells (* p < 0.05, ** p < 0.01, *** p < 0.001).

Figure 2 shows the flow cytometric results of 5 independent experiments using cells from donors of group medium responders for antigen-specific T-cell proliferation in response to HSP70, CMVpp65_495-503 _peptide, and HSP70/CMV-PC. In Figure 2A the results of proliferation of CD8^+ ^T cells are shown, whereas in Figure 2B the results of proliferation of A2/CMV-pentamer-positive CD8^+ ^T cells are shown. PBMCs (19.46% ± 9.05 CD8^+ ^T cells, 0.96% ± 0.56 A2/CMV-pentamer-positive CD8^+ ^T cells) were labeled with CFSE and stimulated with HSP70, CMVpp65_495-503 _peptide, and HSP70/CMV-PC, respectively. The lowest proliferation of CD8^+ ^T cells was observed on day 4 for cells stimulated with HSP70 alone (3.14% ± 0.89%, Figure 2A). Proliferation of CD8^+ ^T cells was significantly higher in cells cultured in the presence of HSP70/CMV-PC (25.09% ± 8.37%) as compared to CMVpp65_495-503 _peptide (6.11% ± 2.71%) alone. The increase in proliferation was even more pronounced when analyzing the proliferation of A2/CMV-pentamer-positive CD8^+ ^T cells (Figure 2B) in the different culture conditions. Again stimulation of cells with HSP70/CMV-PC (13.75% ± 3.67%) resulted in a significantly higher increase of CMV-specific T cells as compared to stimulation with CMVpp65_495-503 _peptide (2.92% ± 1.22%) or HSP70 (0.71% ± 0.47%).

To determine whether the induction of antiviral T cells after stimulation with HSP70/CMV-PC was the result of cross-presentation of chaperoned peptides, the uptake of the complex by the CD91 receptor was blocked with A2M. On day 0, the frequency of CD91 expression on PBMCs of the 5 donors was 26.10% ± 10.92% and of CD40 expression 23.13% ± 16.87%. Addition of A2M to isolated PBMCs significantly reduced the proliferation of CD8^+ ^T cells (Figure 2A) as well as A2/CMV-pentamer-positive CD8^+ ^T cells (Figure 2B) in response to HSP70 and HSP70/CMV-PC. Incubation with A2M prior to stimulation with the complex resulted in a reduced proliferation of 86.86% (from 25.09% ± 8.37 to 3.30% ± 1.45) for CD8^+ ^T cells and of 87.28% (from 13.75% ± 3.67 to 1.75% ± 0.18) for A2/CMV-pentamer-positive CD8^+ ^T cells. The proliferation of T cells in unstimulated cultures as well as CMVpp65_495-503 _peptide-induced T cells remains unaffected. Proliferation of CMV-specific T cells is induced by presentation of the viral peptide chaperoned by HSP70 and can be blocked by adding A2M, the natural ligand of CD91.

### Real-time RT-PCR assessment of target-dependent HSP70 and HO-1 mRNA levels

**Figure 3:**
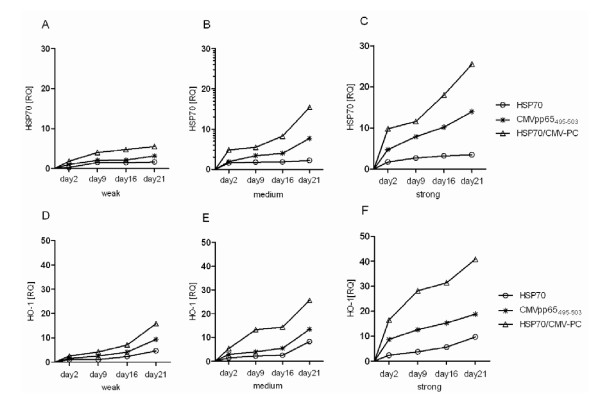
**mRNA expression levels of HSP70 and HO-1 determined by quantitative real-time RT-PCR**. mRNA expression levels of HSP70 (A-C) and HO-1 (D-F) for antigen-specific T cells of donors divided in weak (A, D), medium (B, E), and strong (C, F) after stimulation with HSP70, CMVpp65_495-503 _peptide, and HSP70/CMV-PC were determined by real-time RT-PCR. Constitutively expressed GAPDH gene was used as the reference standard for normalization of mRNA levels. Expression levels of mRNA were measured every second day after stimulation/restimulation and, finally, on day 21. The results of independent experiments are expressed as means (weak: n = 5, medium: n = 5, strong: n = 6). RQ values were calculated by the delta-delta CT method.

In order to determine whether the expression of HSP70 and HO-1 is affected by recombinant HSP70, CMVpp65_495-503 _peptide, and HSP70/CMV-PC we measured levels of HSP70 and HO-1 mRNA by real-time PCR at various time points (Figure 3). Unstimulated T cells were used as negative control and the relative quantification (RQ) values for these experiments were adjusted to 1.00. HSP70 (Figure 3A-C) and HO-1 (Figure 3D-F) mRNA levels were up-regulated in response to HSP70, CMVpp65_495-503 _peptide, and HSP70/CMV-PC in all 3 groups of tested donors. Stimulation with HSP70 caused an induction of the mRNA levels of HSP70 (Figure 3A-C) from day 2 to day 21 (weak: 0.25 to 1.61, medium: 1.58 to 2.21, strong: 1.70 to 3.44). Stimulation with the HSP70/CMV-PC lead to a significant induction of mRNA levels (weak: 1.78 to 5.48, medium: 4.84 to 15.41, strong: 9.80 to 25.50) compared to stimulation with the CMVpp65_495-503 _peptide (weak: 0.99 to 3.12, medium: 1.91 to 7.70, strong: 4.77 to 13.97) alone. Likewise, HO-1 mRNA (Figure 3D-F) levels in the groups of weak/medium/strong responders were 1.02 to 4.65/1.34 to 8.25/2.29 to 9.67 in response to HSP70 and increased significantly in response to CMVpp65_495-503 _peptide (weak: 1.52 to 9.35, medium: 2.85 to 13.42, strong: 8.66 to 18.75) and HSP70/CMV-PC (weak: 2.49 to 15.82, medium: 5.32 to 25.58, strong: 16.41 to 40.81). In general, in comparison to peptide stimulation levels of HSP70 and HO-1 mRNA were significantly higher in all experiments in the HSP70/CMV-PC-stimulated T cells.

### Stimulation with HSP70/CMV-PC up-regulates early IFN-γ and granzyme B secretion

**Figure 4:**
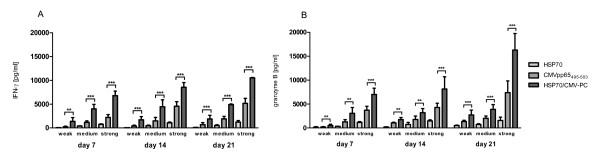
**Detection of IFN-γ and granzyme B protein secretion by ELISA**. The capacity of T cells to secrete cytokines and effector molecules was assessed by determining the secretion levels of (A) the Th type 1 (Th1) cytokine IFN-γ and (B) granzyme B by ELISA. After stimulation with HSP70, CMVpp65_495-503 _peptide, and HSP70/CMV-PC the T-cell culture supernatants were harvested on days 7, 14, and 21 and used for analysis. The results of independent experiments are expressed as means (weak: n = 5, medium: n = 5, strong: n = 6) are expressed as mean ± SD. Asterisks shown in the Figure indicate only statistically significant differences between levels in CMVpp65_495-503 _peptide- and HSP70/CMV-PC-stimulated cells (* p < 0.05, ** p < 0.01, *** p < 0.001).

Secretion levels of the Th1 cytokine IFN-γ (Figure 4A) and the effector molecule granzyme B (Figure 4B) were measured in the supernatant of cells stimulated with HSP70, CMVpp65_495-503 _peptide, or HSP70/CMV-PC by ELISA on days 7, 14, and 21. Unstimulated PBMCs did not secrete high amounts of IFN-γ or granzyme B. The highest amounts of IFN-γ and granzyme B were detected in supernatants of cells stimulated with HSP70/CMV-PC. From day 7 to day 21 the IFN-γ levels of PBMCs stimulated with HSP70 (weak: 35.0 to 91.5 pg/ml, medium: 413.9 to 505.1 pg/ml, strong: 752.3 to 1224.1 pg/ml), CMVpp65_495-503 _peptide (weak: 220.8 to 687.4 pg/ml, medium: 1168.2 to 1906.6 pg/ml, strong: 2206.3 to 5153.2 pg/ml), and HSP70/CMV-PC (weak: 1372.0 to 1890.0 pg/ml, medium: 3985.8 to 4883.9 pg/ml, strong: 6768.7 to 10527.1 pg/ml) were significantly higher than those in unstimulated PBMCs. An increase in granzyme B secretion from day 7 to day 21 was observed in cells cultured in the presence of HSP70 (weak: 169.0 to 506.0 pg/ml, medium: 336.8 to 732.7 pg/ml, strong: 1169.9 to 1572.7 pg/ml), CMVpp65_495-503 _peptide (weak: 205.9 to 1341.1 pg/ml, medium: 1276.3 to 2011.3 pg/ml, strong: 3715.5 to 7384.3 pg/ml), and HSP70/CMV-PC (weak: 503.6 to 2723.4 pg/ml, medium: 3060.7 to 3881.9 pg/ml, strong: 7015.89 to 16288.8 pg/ml).

Interestingly, compared to peptide-stimulated cells in HSP70/CMV-PC-induced T cells a significantly higher secretion of IFN-γ and granzyme B was determined.

## Discussion

In this study, LPS-free recombinant human HSP70 was used to increase the antiviral T-cell response to CMVpp65_495-503 _peptide, the well-known HLA-A*02:01-restricted peptide. For this purpose, HSP70/CMV-PC generated *in vitro *was used to stimulate cytotoxic T cells from unfractionated PBMCs of HLA-A*02:01/CMV-seropositive donors. Our data suggest that linking HSP70 to the CMVpp65_495-503 _peptide can increase antiviral CD8^+ ^T-cell activation and induces a more active phenotype compared to CMVpp65_495-503 _peptide alone. Previous studies have described the adjuvant effects of HSPs following antigen association during the induction of anti-tumor activity [36,37]. Our study is the first to demonstrate that human HSP70 complexed with immunodominant HLA-A*02:01-restricted CMVpp65 peptide (CMVpp65_495-503_) enhances specific antiviral CD8^+ ^CTL responses, especially in donors with low memory CTL precursor frequencies. This strategy opens the stage for GMP-conform improvements of adoptive immunotherapeutic protocols.

CMV infections are a major complication following HSCT and associated with high morbidity and mortality. Adoptive immunotherapy with antigen-specific T cells appears to be a promising treatment for reconstitution of anti-CMV immunity. Therefore, major efforts have been made to identify essential immunogenic CMV-derived epitopes to generate sufficient amounts of antiviral T cells for adoptive transfer. In recent years, two CMV proteins - phosphoprotein 65 (pp65) and immediate-early protein-1 (IE-1) - were found to be major immunodominant targets for the induction of antiviral CD4^+ ^and CD8^+ ^T-cell responses and are considered as candidates for vaccine design [21,40]. About 70% of CMV-specific CTLs recognize pp65-derived epitopes presented by HLA class I molecules [41]. We utilized the immunogenic HLA-A*02:01-restricted CMVpp65_495-503 _peptide [29,42] as a viral target and observed that cross-presentation of this peptide by recombinant HSP70 yielded in a significantly higher number of antigen-specific T cells compared to the use of the CMVpp65_495-503 _peptide alone. Expression of soluble HSP70 in mammalian cells was recently established to prevent pathogen-associated molecular pattern (PAMP) contamination [35].

Several studies have shown that HSP/PCs can induce efficient cell-mediated immunity to human tumor antigens and improve the frequency of antigen-specific cytotoxic CD8^+ ^T cells [43-45]. It is known, that the adaptive immune response is initiated by receptor-mediated endocytosis of the HSP/PCs (Additional file 2), which deliver the peptides via both cytosolic and endocytic routes of antigen processing for cross-presentation in MHC class I molecules on the surface of APCs [8,44,46,47]. So far, only CD91 [14] has been shown to be a key receptor for the uptake and cross-presentation of HSP70/PCs. We likewise observed that A2M, which acts as a natural ligand for CD91 [46,48], strongly inhibits the proliferation of antiviral T cells stimulated with HSP70/CMV-PC. When inhibition was ≤ 86.86% (CD8^+ ^T cells) and ≤ 87.26% (A2/CMV-pentamer-positive CD8^+ ^T cells), the proliferation capacity of antiviral T cells was not completely blocked. These findings indicate that the uptake of HSP70/PCs is mainly mediated by CD91, but other independent receptor systems of APCs could also be involved. Here CD40- [19] and LOX-1-mediated uptake [18,49] might play a role.

The frequency of antiviral CD8^+ ^T cells increased significantly in all stimulation groups, but varied depending on the individual donor PBMCs. It is well known that the frequencies of CMV-specific T cells can range from roughly 2 to 10 percent to more than 70 to 90 percent, even in individuals who are clearly responders [31]. We demonstrated that only 52.00% of 50 HLA-A*02:01/CMV-seropositive donors were A2/CMV-pentamer-positive. The variable range of A2/CMV-pentamer-positive CD8^+ ^T-cell frequencies from 0.30% to 6.70% in our test subjects emphasize the necessity to classify potential donors. Interestingly, we found that the number of CMV-specific memory T cells present does not correlate with the determined increase in antigen-specific T-cell frequencies after stimulation with neither the peptide alone nor the complex. Therefore, we decided to classify the 16 HLA-A*02:01/CMV-seropositive donors as weak, medium, and strong responder according to the frequency of generated A2/CMV-pentamer-positive CD8^+ ^T cells after 1 week stimulation with the well-known CMVpp65_495-503 _peptide. Donors classified in these groups belonged there for the whole stimulation period.

The levels of antiviral CD8^+ ^as well as CD4^+ ^T-cell responses depend on the peptide. IE-1 and CMVpp65 have been recognized as source of immunodominant antigens that stimulate both cytotoxic and T-helper cells. HLA class I-restricted peptides derived from these proteins are known to be potent inducers of CTLs [29,31,32,50], but the obtained responses against CMVpp65 peptides are in general stronger than those described for IE-1-derived peptides [40,51]. Lacey *et al. *studied CD8^+ ^T-cell responses against three human CMVpp65 epitopes in healthy CMV-seropositive donors [52]. A significant response was observed to the HLA-A*02-restricted epitope within the CMVpp65 antigen for HLA-A*02:01-positive donors which do not express the HLA-B*07:02 allele. By contrast, the strongest responses to CMV in the group of HLA-A*02:01/HLA-B*07:02-positive donors were to HLA-B*07-restricted epitopes, indicating that the HLA-B*07:02-restricted T-cell response was shown to be dominant over HLA-A*02:01. Here we focused on the HLA-A*02:01-restricted CMVpp65 peptide to evaluate the effect of cross-presentation in the expansion of antiviral T-cells and hypothize, that the observed effects can be retransmitted to several viral peptides. Cross presentation of tumor-derived peptides by HSP70 as well as by gp96 was shown before to increase the immune response [20,47,48,53]. To further study the underlying mechanism we performed T-cell stimulation using HSP70 complexed with either A*02:01_CMV-IE-1_81-89 _(n = 5 donors), A*01:01_CMVpp65_363-373 _(n = 5 donors), or B*07:02_CMVpp65_417-426 _(n = 4 donors) and expanded these cells over 3 weeks with the described protocol (Additional file 3). In all experiments we found a strong increase in the antiviral T-cell response using the respective HSP70/PC compared to stimulation with the viral peptides alone. These data support the findings described in this manuscript.

To assess the activation status of antiviral T cells generated *in vitro *we measured expression levels of T-cell activation markers such as CD25, CD69, and CD137 on the cell surface [54,55]. Expression levels of all activation markers used in the present study increased after stimulation. HSP70/CMV-PC induced the highest frequency of CD25, CD69, and CD137 expression in CD8^+ ^T cells as well as in A2/CMV-pentamer positive CD8^+ ^T cells.

In this study, we also determined the mRNA expression levels of HSP70 and HO-1 in activated T cells by quantitative real-time RT-PCR. HSP70 and HO-1, both of which are inducible cytoprotective stress proteins, have previously been shown to be up-regulated in response to similar stimuli [56,57]. But, in particular, the regulation of HO-1 expression in T cells is not well studied and contradictory results have been reported. Stimulation with HSP70/CMV-PC caused a significantly higher HSP70 and HO-1 mRNA expression levels than either HSP70 or CMVpp65_495-503 _peptide alone. Interestingly, mRNA levels of HO-1 were significantly higher compared to that of HSP70. HO-1 is generally known to be induced by cellular stress and has major antioxidant and anti-inflammatory functions [58]. Biburger suggests that HO-1 may modulate the proliferative capacity of T lymphocytes [58]. Another study unexpectedly demonstrated the activity of HO-1 in human cancer cell during tumor progression [59]. Here, we demonstrate for the first time that the HO-1 expression level is up-regulated in specific CD8^+ ^and CD4^+ ^T cells after stimulation with viral antigens or HSP70, whereas HSP70/CMV-PC can significantly increase HO-1 expression.

Compared to unstimulated cells, a slight increase of secretion of granzyme B was observed after stimulation with HSP70 alone, which is in concordance with previous findings [35]. Still stimulation with HSP70/CMV-PC resulted in the highest granzyme B concentrations seen.

So far little is known about the "peptide binding motif" of HSP70 [60]. Therefore, at least three hypotheses have been proposed to explain the affinity of peptides to HSP70: 1) Dependent on the hydrophobic residues of the peptide [19] and on the ATP/ADP-bound state of HSP70, the peptide binding may change the conformation and rigidity of HSP70, potentially altering the choice of receptors on APCs, which will be used by the respective HSP70/PCs [19]; 2) Higher affinity may affect peptide processing within the cells by increasing the half-life as the peptide is protected from proteolytic digestion [61]; 3) By increasing interaction with the receptor in this manner, a greater proportion of HSP70 is bound by the peptide [19]. So far for efficient induction of antigen-specific cells, HSP70/PC concentrations have been higher than 50 μg per stimulation [62]. We achieved similar antigen-specific T-cell responses using only one-tenth of the typical amount of HSP70/PCs, which has both material and cost advantages and might indicate that by using immunodominant peptides with a high affinity to HSP70, low concentrations of the antigenic peptide may suffice to achieve significant increases in antiviral T-cell responses. However, the analysis of the spin column flow confirm, that up to 10 μg/ml HSP70/CMV-PC was generated. Still, the exact concentrations of low-affinity or weak peptides needed to evoke significant specific T-cell responses remains to be proven.

## Conclusions

In summary, we were able to demonstrate that human antiviral CTLs can be successfully generated *in vitro *using the HSP70/CMV-PC. The intact effector function of the induced CTLs was demonstrated by functional assays. Stimulation with HSP70/PCs leads to early production of effector cytokines. These findings are consistent with the concept that HSP70/PCs result in efficient cross-presentation by HLA class I molecules and in significantly higher antigen-specific T-cell responses than non-complexed immunodominant peptides.

Our results clearly indicate that HSP70/CMV-PC can act as potent mediator for the *in vitro *generation of the amounts of antigen-specific T cells needed for adoptive immunotherapy. Especially in cases of naïve or low CTL precursor frequencies HSP70-chaperoned peptides might be useful in clinical applications including the selective induction of T cells directed against leukemia targets to increase the graft-versus-leukemia effect, e.g. by using minor histocompatibility antigen-specific T cells and the selective expansion of T cells against viral targets to increase the graft-versus-infection effect. This approach seems to be a promising method to improve clinical outcome in children, who have the highest rates of adenovirus infection, which is associated with high morbidity and mortality, and where less cells are available for T-cell induction.

The method for production of recombinant human HSP70 can be adapted to GMP conditions and can be used to generate the large amounts of immunogenic HSP70/peptide or protein complexes needed to generate antigen-specific T cells for clinical applications.

## Competing interests

The authors declare that they have no competing interests.

## Authors' contributions

ST participated in the design of the study, carried out the experiments, performed the statistical analysis and drafted the manuscript. MB participated in the experiments for T-cell stimulation. BMK, SI, MO, and RB contributed with helpful discussion and helped to draft the manuscript. BEV designed this study, participated in the statistical analysis, and drafted the manuscript. All authors read and approved the final manuscript.

## Supplementary Material

Additional file 1 **Flow cytometric analysis of antigen-specific T cells stimulated with HSP70/CMV-PC and CMVpp65_495-503 _peptide**. Frequency of A2/CMV-pentamer-positive CD8^+ ^T cells on day 0 and 7, 14 and 21 days after stimulation with recombinant HSP70, respective CMVpp65_495-503 _peptide, and HSP70/CMV-PC. Cells cultured in the presence of the HSP70-peptide-binding buffer served as negative controls (NC). The donors were divided into three groups (weak: n = 5, medium: n = 5, strong: n = 6) according to the frequency of generated A2/CMV-pentamer-positive CD8^+ ^T cells on day 7 (Table 1). Shown are representative results each with one donor from the group of weak (A), medium (B), or strong (C) responder. Click here for file

Additional file 2 **Analysis of HSP70/CMV[FITC]-PC, CMVpp65_495-503 _peptide-FITC, and HSP70-FITC uptake by T-cell subsets using immunfluorescence microscopy**. Representative results of immunfluorescence microscopy of isolated monocytes from HLA-A*02:01-positive healthy donor (purity > 98%, Monocyte Isolation Kit II, Miltenyi Biotech, Bergisch Gladbach, Germany). Immunofluorescence assay was performed as described elsewhere by Bajor *et al. *[62]. For the experiment, 33 mg mg purified HSP70 were conjugated with FITC (Fluoro tag FITC conjugation Kit, Sigma-Aldrich, Hamburg, Germany) as well as using FITC-labeled HLA-A*02:01-restricted CMVpp65_495-503 _peptide (CMV[FITC], GL Biochem) in a complex with HSP70 (HSP70/CMV[FITC]-PC). Therefore additional amino acid lysine (K) in the peptide sequences was required for FITC-labeling of the peptide HSP70/CMV[FITC]-PC and was prepared as described for the unlabeled complex. 1 × 10^6 ^monocytes were incubated for 4 h with either (A) alone, (B) 10 μg/ml HSP70/CMV[FITC]-PC, (C) 10 μg/ml FITC-labeled HSP70 (HSP70-FITC), or (D) 10 μg/ml FITC-labeled CMVpp65_495-503 _peptide (CMVpp65_495-503 _peptide-FITC) in 500 μl culture medium (37°C) using 4 well chamber slides (Sigma-Aldrich, Ontario, Canada). Incubation of CD14^+ ^cells with (B) HSP70/CMV[FITC]-PC resulted in an increase of uptake in comparison to (C) HSP70-FITC or (D) CMVpp65_495-503 _peptide-FITC alone. Analysis was performed on the Olympus-IX81 microscope (Olympus, PA, USA) with a DAPI and FITC filter set using a 40X objective. Images were acquired using a CCD camera (Olympus) and analyzed using Olympus cell^IM ^and cell^IR ^image 3.0 software (Olympus). Click here for file

Additional file 3 Pentamer staining of antigen-specific CTLs stimulated with A*02:01_CMV-IE-1_81-89_, A*01:01_CMVpp65_363-373_, or B*07:02_CMVpp65_417-426 _peptides and the respective HSP70/peptide complexes. Frequency of A2/CMV-IE-1_81-89_-pentamer-positive CD8^+ ^T cells (A, n = 5), A1/CMVpp65_363-373_-pentamer-positive CD8^+ ^T cells (B, n = 5), and B7/CMVpp65_417-426_-pentamer-positive CD8^+ ^T cells (C, n = 4) from healthy A2/CMV-pentamer-positive donors on day 0 and 7, 14 and 21 days after stimulation with recombinant HSP70, the respective HLA-restricted CMV peptide or the respective HSP70/PC. Cells cultured in the presence of the HSP70-peptide-binding buffer served as negative controls (NC). The donors were divided into three groups (weak, medium, strong) according to the frequency of generated CMV-pentamer-positive CD8^+ ^T cells on day 7. The results of the independent experiments for all donors are expressed as mean ± SD (no division of the donors in groups). Asterisks shown in the Figure indicate statistically significances between levels in CMV peptide- and respective HSP70/PC-stimulated cells (*p < 0.05, ** p < 0.01). Click here for file
