# Supplementary material for: Heat shock protein 70/peptide complexes: potent mediators for the generation of antiviral T cells particularly with regard to low precursor frequencies
Source: J Transl Med. 2011 Oct 12;9:175. doi: 10.1186/1479-5876-9-175 (PMC3217864; doi:10.1186/1479-5876-9-175)
Supplement: Additional file 1 — Flow cytometric analysis of antigen-specific T cells stimulated with HSP70/CMV-PC and CMVpp65495-503 peptide. Frequency of A2/CMV-pentamer-positive CD8+ T cells on day 0 and 7, 14 and 21 days after stimulation with recombinant HSP70, respective CMVpp65495-503 peptide, and HSP70/CMV-PC. Cells cultured in the presence of the HSP70-peptide-binding buffer served as negative controls (NC). The donors were divided into three groups (weak: n = 5, medium: n = 5, strong: n = 6) according to the frequency of generated A2/CMV-pentamer-positive CD8+ T cells on day 7 (Table 1). Shown are representative results each with one donor from the group of weak (A), medium (B), or strong (C) responder. [file 1479-5876-9-175-S1.DOC]

**A) weak responder (donor 4)**

**day 0**

0.37%

**day 7**

CMVpp65_495-503_

HSP70/CMV-PC

NC

HSP70

0.30%

0.70%

0.38%

0.50%

**
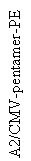
**

**day 14**

6.40%

0.93%

0.70%

0.40%

**day 21**

0.70%

0.79%

5.30%

14.60%

CD8-APC

**B) medium responder (donor 6)**

**day 0**

0.30%

**day 7**

CMVpp65_495-503_

HSP70/CMV-PC

NC

HSP70

4.50%

0.30%

0.40%

9.50%

**
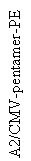
**

**day 14**

32.80%

53.90%

0.40%

0.70%

**day21**

2.20%

2.20%

64.00%

47.80%

CD8-PerCP

**C) strong responder (donor 13)**

**day 0**

1.40%

**day 7**

CMVpp65_495-503_

HSP70/CMV-PC

NC

HSP70

**
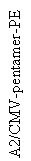
**

0.40%

0.71%

21.00%

44.50%

**day 14**

89.35%

52.00%

0.70%

0.80%

**day21**

2.20%

0.90%

72.81%

97.83%

CD8-APC
