# Supplementary figures and images for: Heat shock protein 70/peptide complexes: potent mediators for the generation of antiviral T cells particularly with regard to low precursor frequencies
Source: J Transl Med. 2011 Oct 12;9:175. doi: 10.1186/1479-5876-9-175 (PMC3217864; doi:10.1186/1479-5876-9-175)

**A b C D**

**
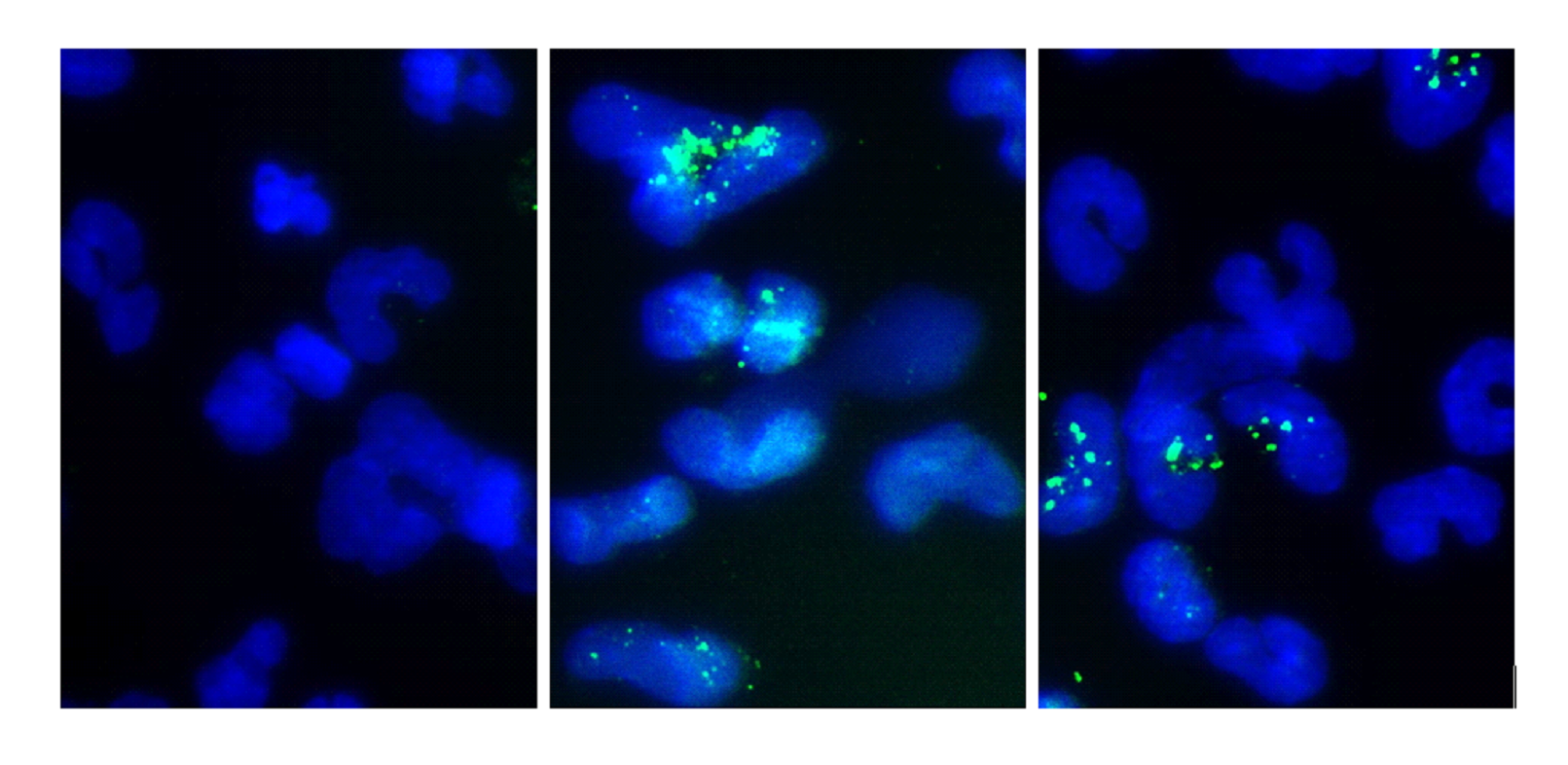
**
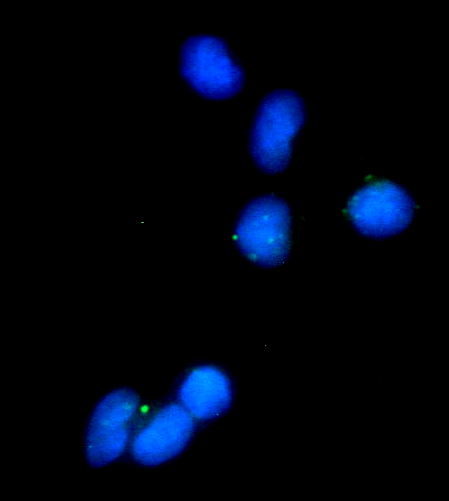

Supplement: Additional file 2 — Analysis of HSP70/CMV[FITC]-PC, CMVpp65495-503 peptide-FITC, and HSP70-FITC uptake by T-cell subsets using immunfluorescence microscopy. Representative results of immunfluorescence microscopy of isolated monocytes from HLA-A*02:01-positive healthy donor (purity > 98%, Monocyte Isolation Kit II, Miltenyi Biotech, Bergisch Gladbach, Germany). Immunofluorescence assay was performed as described elsewhere by Bajor et al. [62]. For the experiment, 33 mg mg purified HSP70 were conjugated with FITC (Fluoro tag FITC conjugation Kit, Sigma-Aldrich, Hamburg, Germany) as well as using FITC-labeled HLA-A*02:01-restricted CMVpp65495-503 peptide (CMV[FITC], GL Biochem) in a complex with HSP70 (HSP70/CMV[FITC]-PC). Therefore additional amino acid lysine (K) in the peptide sequences was required for FITC-labeling of the peptide HSP70/CMV[FITC]-PC and was prepared as described for the unlabeled complex. 1 × 10^6^ monocytes were incubated for 4 h with either (A) alone, (B) 10 μg/ml HSP70/CMV[FITC]-PC, (C) 10 μg/ml FITC-labeled HSP70 (HSP70-FITC), or (D) 10 μg/ml FITC-labeled CMVpp65495-503 peptide (CMVpp65495-503 peptide-FITC) in 500 μl culture medium (37°C) using 4 well chamber slides (Sigma-Aldrich, Ontario, Canada). Incubation of CD14+ cells with (B) HSP70/CMV[FITC]-PC resulted in an increase of uptake in comparison to (C) HSP70-FITC or (D) CMVpp65495-503 peptide-FITC alone. Analysis was performed on the Olympus-IX81 microscope (Olympus, PA, USA) with a DAPI and FITC filter set using a 40X objective. Images were acquired using a CCD camera (Olympus) and analyzed using Olympus cellIM and cellIR image 3.0 software (Olympus). [file 1479-5876-9-175-S2.DOC]
