# Supplementary material for: Heat shock protein 70/peptide complexes: potent mediators for the generation of antiviral T cells particularly with regard to low precursor frequencies
Source: J Transl Med. 2011 Oct 12;9:175. doi: 10.1186/1479-5876-9-175 (PMC3217864; doi:10.1186/1479-5876-9-175)
Supplement: Supplementary file 3 [file 1479-5876-9-175-S3.DOC]

A

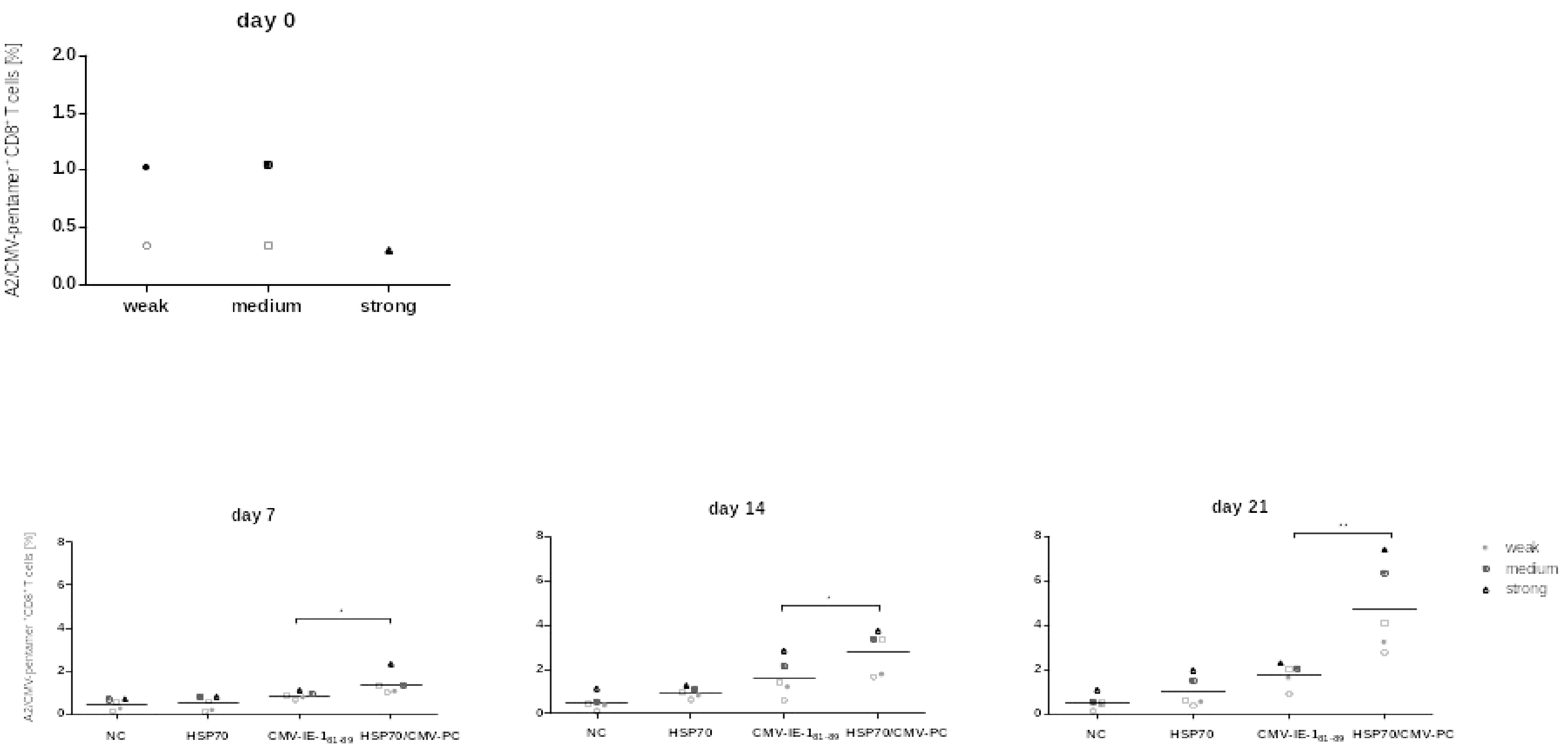
day 0
A2/CMV-pentamer⁺ CD8⁺ T cells [%]
2.0
1.5
1.0
0.5
0.0
weak
medium
strong
day 7
day 14
day 21
8
6
4
2
0
NC
HSP70
CMV-IE-1 81-89
HSP70/CMV-PC
weak
medium
strong

B

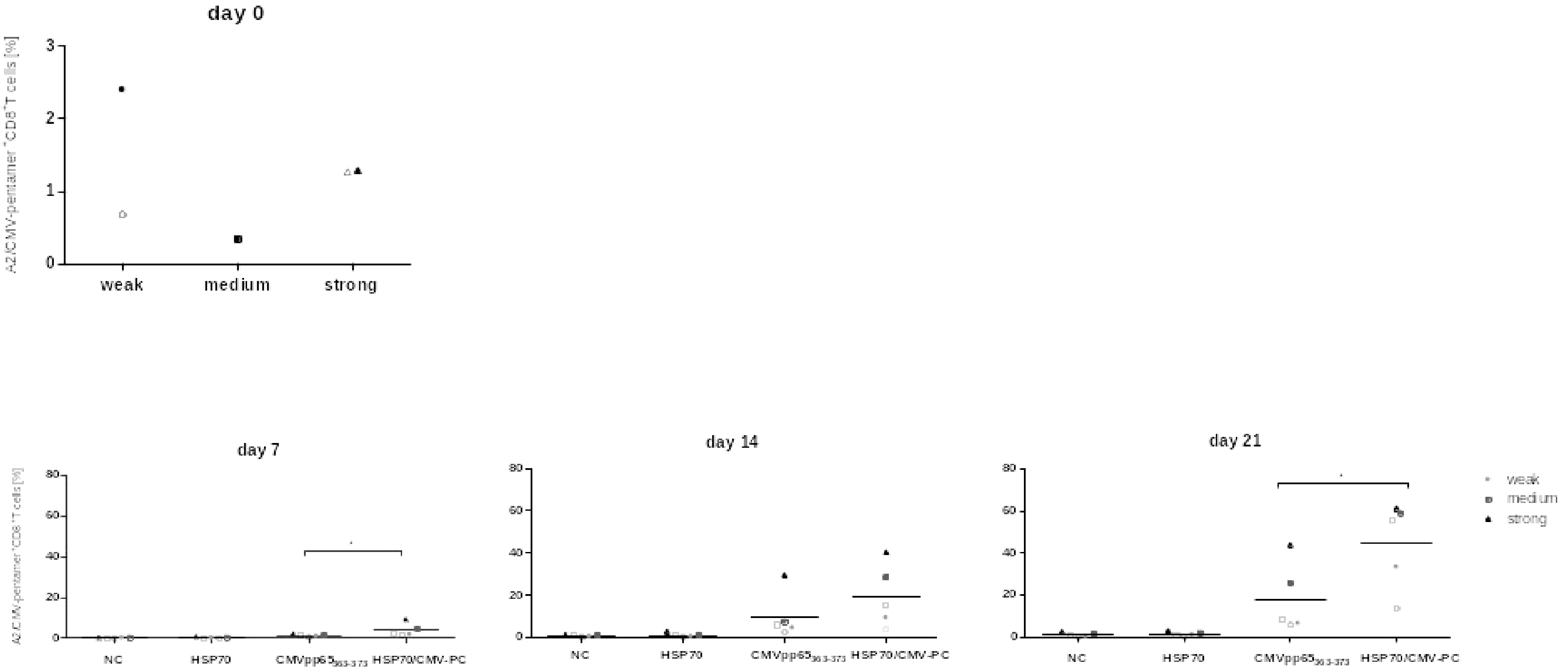
day 0
A2/CMV-pentamer⁺CD8⁺T cells [%]
3
2
1
0
weak
medium
strong
day 7
day 14
day 21
80
60
40
20
0
NC
HSP70
CMVpp65363-373
HSP70/CMV-PC
weak
medium
strong

C

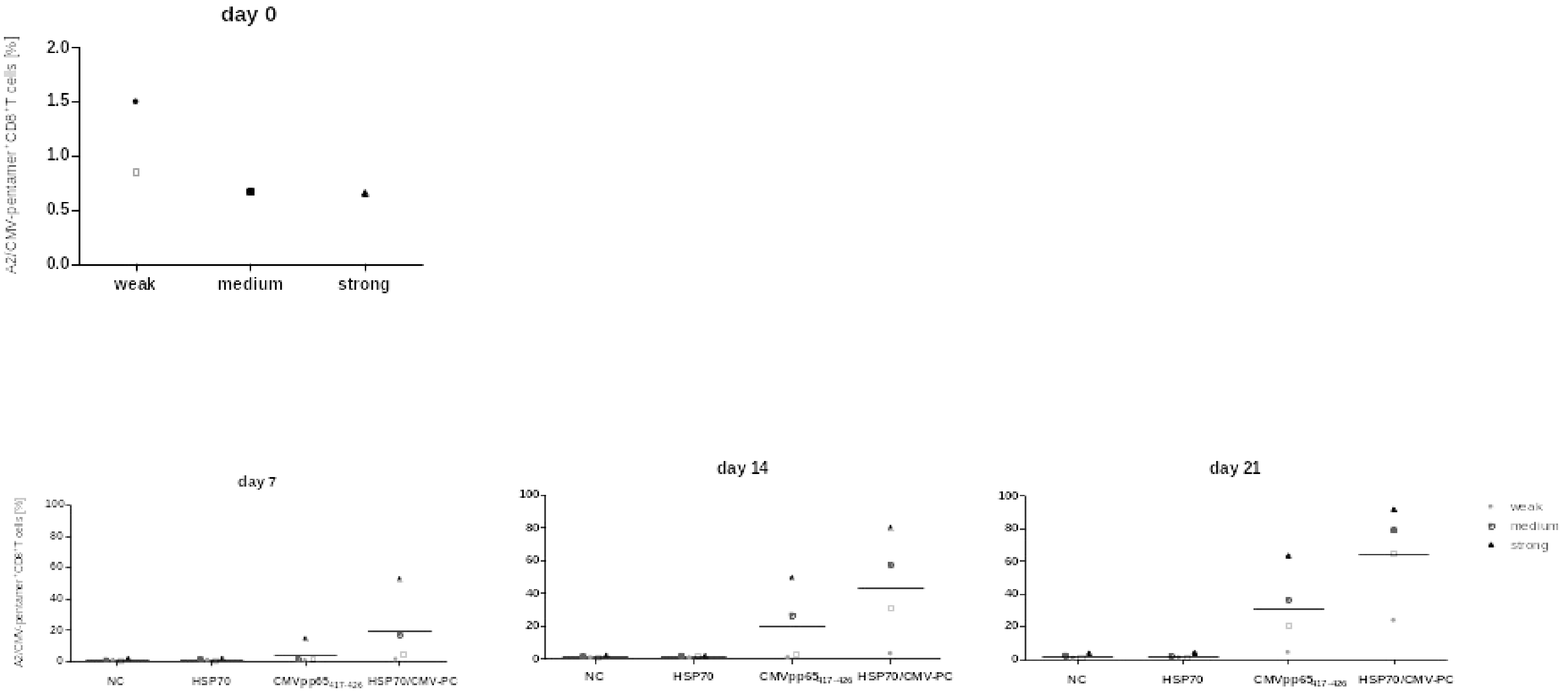
day 0
A2/CMV-pentamer⁺CD8⁺T cells [%]
2.0
1.5
1.0
0.5
0.0
weak
medium
strong
day 7
day 14
day 21
100
80
60
40
20
0
NC
HSP70
CMVpp65417-426
HSP70/CMV-PC
weak
medium
strong
